# Effect of exercise versus cognitive behavioural therapy or no intervention on anxiety, depression, fitness and quality of life in adults with previous methamphetamine dependency: a systematic review

**DOI:** 10.1186/s13722-018-0106-4

**Published:** 2018-01-16

**Authors:** Linzette Morris, Jessica Stander, Wardah Ebrahim, Stephanie Eksteen, Orissa Anna Meaden, Ané Ras, Annemarie Wessels

**Affiliations:** Division of Physiotherapy, Department of Health and Rehabilitation Sciences, Faculty of Medicine and Health Sciences, Stellenbosch University, PO Box 241, Cape Town, 8000 South Africa

**Keywords:** Methamphetamine, Exercise, Fitness, Quality of life, Depression, Anxiety

## Abstract

**Background:**

Methamphetamine (MA) is a highly addictive psychostimulant used by approximately 52 million people worldwide. Chronic MA abuse leads to detrimental physiological and neurological changes, as well as increases in anxiety and depression, and decreases in overall fitness and quality of life. Exercise has been reported to possibly reverse physiological and neurological damage caused by previous MA use, and to reduce anxiety and depression in this population. The aim of this systematic review was to identify, clinically appraise and synthesise the available evidence for the effectiveness of exercise, compared to cognitive behavioural therapy (CBT), standard care or no intervention, on decreasing anxiety and depression and improving fitness and quality of life in previous MA users.

**Methods:**

Seven computerised databases were searched from inception to May 2017, namely Scopus, Cochrane Library, PubMed/MEDLINE, PEDro, CINAHL, and ScienceDirect. Search terms included exercise, methamphetamine, fitness measures, depression, anxiety and quality of life. Randomised and non-randomised controlled- or clinical trials and pilot studies, published in English, were considered for inclusion. Methodological quality was critically appraised according to the PEDro scale. Heterogeneity across studies regarding control groups and assessment intervals rendered meta analyses inappropriate for this review and results were thus described narratively using text and tables.

**Results:**

Two hundred and fifty-one titles were identified following the initial search, and 14 potentially-relevant titles were selected and the abstracts reviewed. Three studies (two randomised controlled trials and one quasi-experimental pilot) were included, with an average PEDro score of 6.66. Exercise resulted in significantly lower depression and anxiety scores versus CBT (p = 0.001). Balance also significantly improved following exercise versus standard care (p < 0.001); as did vital capacity, hand-grip and one-leg stand with eyes closed. There were significant changes in all subdivisions of the Quality of Life Scale Questionnaire (p < 0.05), except psychology (p = 0.227).

**Conclusions:**

Level II evidence suggests that exercise is effective in reducing anxiety and depression and improving fitness in previous MA users, and Level III-2 evidence suggests that exercise is beneficial for improving quality of life in this population. Overall recovery in previous MA dependents might be significantly enhanced by including exercise in the rehabilitation process. Further research is required to strengthen these conclusions and to inform policy and health systems effectively.

**Electronic supplementary material:**

The online version of this article (10.1186/s13722-018-0106-4) contains supplementary material, which is available to authorized users.

## Background

Methamphetamine (MA) is a psychostimulant substance which causes various physiological effects in the body and brain, and is highly addictive [1]. It is estimated that MA substance abuse disorder, generally indicated as ICD-10 code F15.20 (DSM-5 code 304.40) [2], affects nearly 52 million people worldwide, and is the second most widely used drug of its kind in countries like North and South America, etc. [3–5]. Typically used among younger populations, people who abuse MA may begin to suffer from depression, psychosis, behavioral disorders which may eventually lead to suicide and overdose [5]. The abuse of MA has serious ramifications for the individual, society, the economy, the environment and the health system of a country [5, 6]. The high prevalence of MA use among populations in various countries, especially among younger populations, and the resulting consequences of the drug on the health on the individual is therefore a major global concern [3–6].

In the brain, MA binds to dopamine, nor-epinephrine and serotonin transporters on the neuronal cells and stimulates the fight-or-flight response via excessive stimulation of the sympathetic nervous system [7, 8]. Using MA in the short-term MA therefore improves productivity, attention-span and energy levels, and may even reduce anxiety [9]. In contrast, however, chronic use depletes dopamine stores in the brain and damages the ability of dopamine and serotonin to bind to their terminals [7]. This renders the chronic user incapable of experiencing pleasure naturally and may therefore lead to depression [9]. Another complication of long-term use is impaired neuropsychiatric function, including motor- and executive function as well as episodic memory, which is commonly associated with anxiety and depression [10].

The detrimental effects of excessive MA abuse, like any other synthetic drug, on health and fitness is also well documented [4, 5, 11]. Long-term drug use often leads to chronic conditions such as hypertension, strokes and myocardial infarctions, reduced cardiovascular fitness as well as movement disorders, including reduced balance and flexibility [4, 5, 11]. The individual recovering from MA dependence may therefore be left with not only the fear of relapsing, but also a variety of conditions which may make it difficult to work or even execute activities of daily living [4, 5, 11]. Therefore, in addition to the physical and financial burden post-rehabilitated MA dependents may place on society due to the inability to work, their individual quality of life may be greatly compromised which may lead to further depression and anxiety.

However, contrary to previous beliefs that the physiological and neurological damage caused by drugs were permanent, empirical evidence has shown that the damage caused by drug abuse may actually be reversed through exercise [12, 13]. It has been reported that-aerobic exercise in particular had positive physiological effects on the brain and dopamine and serotonin levels of patients who had used MA [12, 13] by decreasing levels of pro-inflammatory biomarkers in the circulatory system [12, 14, 15]. In addition, it has been proposed that exercise decreases stress, and subsequently anxiety and depression, by activating the adrenal gland activity [15]. Furthermore, resistance training has also proven beneficial in decreasing the neurological effects of MA and improving cardiovascular fitness [16, 17]. Other more well-known effects of exercise include improvements in balance and flexibility and the reduced risk of chronic conditions such as hypertension, strokes and heart attacks [18, 19]. Exercise may therefore be beneficial to treat depression and anxiety, as well as improve overall fitness and essentially quality of life, in previously MA-dependent individuals.

Another possible approach for targeting depression and anxiety, specifically in previous drug users, is the use of cognitive behavioural therapy (CBT). CBT is a comprehensive set of educational and psychological techniques that equips drug users with knowledge about stimulant dependence and teaches them skills to both initiate abstinence, as well as return to abstinence should relapse occur [20]. However, conflicting evidence exists regarding the efficacy of CBT, with some studies concluding that CBT seems ineffective in improving depression and physiological changes among previous MA users [21, 22].

To date, no systematic review has been conducted to determine the effect of exercise compared to CBT, no intervention or standard care, in the management of previously MA-dependent adults suffering from anxiety, depression, decreased fitness and reduced quality of life. The purpose of this systematic review was thus to identify, critically appraise and synthesise current evidence for the effectiveness of exercise versus CBT, no intervention or standard care in decreasing anxiety and depression, improving fitness levels and improving quality of life in previously MA-dependent adults. By doing so, this review aims to provide physiotherapists with evidence-based treatment options for the rehabilitation of previous MA dependents and to confirm the role of physiotherapists in the management of rehabilitating drug users.

## Methods

### Search strategy

Seven computerised bibliographic databases, accessed through the Stellenbosch University library services, were searched, namely Scopus, Cochrane Library, PubMed, PEDro, CINAHL, MEDLINE ProQuest and ScienceDirect. The date limit was initially set from inception up to April 2016. An update of the search was conducted in June 2016, and again in May 2017. Preliminary searches within each database allowed for the elimination of unnecessary search terms, where the addition of keywords did not yield varying results. Key search terms included methamphetamine, exercise, physical activity, depression, anxiety, psychological disorders and fitness measures. The detailed search strategies, specifically developed for each database according its functions, are provided in Additional file 1.

### Review team

The review was performed as part of an undergraduate research project in the Physiotherapy Division at Stellenbosch University, South Africa, under the guidance of LM and JS. The five undergraduate students received training in conducting systematic reviews. The supervisor (LM) is well trained in conducting systematic reviews and also provides training for systematic reviews at all levels (undergraduate and postgraduate level across faculties).

### Study selection

Each of the five reviewers independently searched two randomly-selected databases and screened titles according to the eligibility criteria of the review. Results were subsequently cross-checked between reviewers. Any disagreements were resolved by discussion and consensus between the reviewers, and where no consensus could be reached, the study supervisors were asked to adjudicate. Titles which were obviously not relevant were disregarded, and titles which seemed vaguely relevant were selected. If the reviewer was unsure about a particular title, the abstract was retrieved for that article to review. Duplicate titles were eliminated across database search results. Abstracts for all selected titles were retrieved and each reviewer independently screened the abstracts against the eligibility criteria. Reviewers compared potentially eligible abstracts amongst each other and any disagreements regarding in-/exclusion were resolved by contacting the supervisors. Full-text articles were subsequently retrieved via electronic journals and/or hard copies, and were independently screened for eligibility by each reviewer. The reviewers compared the eligible full-texts identified for inclusion and if consensus regarding final inclusion of articles was not reached, the supervisors were contacted to resolve the matter.

### Criteria for considering studies

#### Types of studies

Randomised controlled- or clinical trials (RCTs), non-randomised controlled- or clinical trials and quasi-experimental studies published in English from inception of the database until May 2017 were considered for inclusion in this review.

#### Types of participants

Study participants had to be adult (> 18 years) male and/or female previous MA users who were involved in rehabilitation at the time of intervention. Studies were excluded if they involved participants who were non-MA drug dependent individuals (for example those using other drugs such as cocaine, heroin, etc.), suffered from severe medical conditions compromising the participant’s safety during physical exercise, were pregnant, or were diagnosed with infectious diseases such as syphilis and hepatitis.

#### Types of interventions

Whole-body exercise programs including, but not confined to, aerobic exercises, resistance exercises, strength training, cardiovascular exercises and Tai Chi were eligible interventions.

#### Types of comparison

Control groups had to receive CBT as either an educational or psychological program, standard care or no intervention.

#### Types of outcome measures

The following outcome measures were considered eligible: (1) anxiety measures including, but not confined to, the Beck Anxiety Inventory (BAI); (2) depression measures including, but not confined to, the Beck Depression Inventory (BDI); (3) fitness measures including, but not confined to, aerobic performance (VO2Max), musculoskeletal fitness [one-repetition maximum method (1-RM) for the leg and bench press exercise], body mass (kg), body mass index (BMI) (kg m^−2^), body fat (%), systolic (mmHg) and diastolic (mmHg) readings, pulse (bpm); vital capacity (ml); Sit-And-Reach (cm), and single leg stance with eyes closed (s); and (4) quality of life measures including, but not confined to, the Quality of Life for Drug Addiction (QOL-DA) Questionnaire.

### Evidence hierarchy

The National Health and Medical Research Council (NHMRC) Evidence Hierarchy [23], presented in Additional file 2, was used to rank the level of evidence of the included studies. This important step ensures reliability and validity of the articles included in a systematic review [23]. Each reviewer independently scored the level of evidence for every article. Results were discussed amongst the reviewers and discrepancies referred to the supervisors to adjudicate.

### Methodological appraisal

The PEDro scale [24] (Additional file 3) was used to score the methodological quality of each included article. The instrument was developed to rate the quality of RCTs and clinical trials on the Physiotherapy Evidence Database and is a widely used, valid and reliable measure [25, 26]. The eleven criteria in the scale, scored as either present (1) or absent (0), sum to a total score of 10 [26]. Reviewers individually scored each article, where after discrepancies were discussed within the review group and resolved by contacting the study supervisors.

### Data extraction

Data were extracted from the included articles using the adapted Joanna Briggs Institute (JBI) data extraction form for the systematic review of experimental and/or observational studies (Additional file 4). Extracted data were organized according to the following categories: citation, study design, participants (including baseline characteristics), outcome measures, interventions (for both treatment- and control groups), results, as well as post-intervention clinical status and the implications thereof. Reviewers performed independent data extraction, with subsequent cross-checking of findings to identify any missing data or errors incurred during the extraction process, and to ensure consensus. In cases where data from articles were missing, the relevant author was contacted via email to request information.

### Data analysis

Following data extraction, each outcome was allocated to a pair of reviewers for analysis. To confirm accuracy, analyses were cross-checked by the rest of the reviewers and the supervisors were contacted for assistance if disagreements arose. Due to substantial heterogeneity amongst studies regarding control groups, outcome measures and assessment intervals, statistical pooling was not appropriate. Results were therefore summarised narratively using text paragraphs and tables.

## Results

### Search results and description of studies

**Fig. 1:**
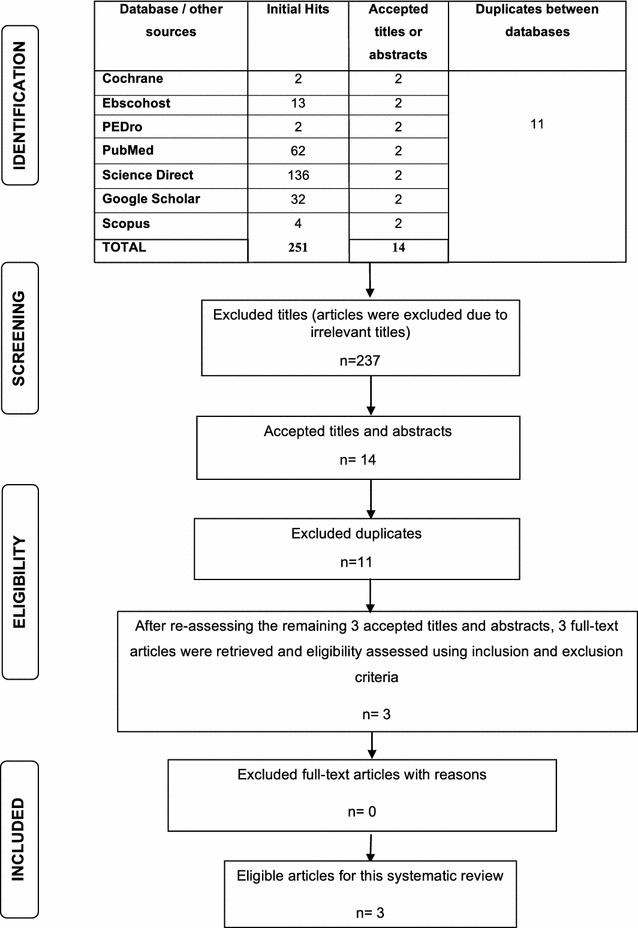
Results of search strategy

A total of 251 titles were identified following the initial search. Of these, 14 accepted titles were reviewed and three full-text articles were subsequently deemed eligible for inclusion in this review. The search process and results (as well as reasons for exclusion) are depicted in Fig. 1.

### Evidence hierarchy

The final three articles that were used in this systematic review included two RCTs [27, 28] and one quasi-experimental pilot study [29]. According to the NHMRC Hierarchy of Evidence [30], the two RCTs were classified as Level II and the quasi-experimental pilot study as Level III-2 evidence.

### Methodological appraisal

The methodological quality of the three included articles was assessed using the 11-item PEDro scale. obtaining an average score of 6.66/11. Table 1 summarises the individual PEDro scores of the articles.

**Table 1:** Methodological quality of included studies

PEDro criteria	Rawson et al. [28]	Dolezal et al. [27]	Zhu et al. [29]
1. Eligibility criteria were specified	√	√	√
2. Subjects were randomly allocated to groups (in a crossover study, subjects were randomly allocated an order in which treatments were received)	√	√	
3. Allocation was concealed	x	x	x
4. The groups were similar at baseline regarding the most important prognostic indicators	√	√	√
5. There was blinding of all subjects	x	x	x
6. There was blinding of all therapists who administered the therapy	x	x	x
7. There was blinding of all the assessors who measured at least one key outcome	x	x	√
8. Measures of at least one key outcome were obtained for more than 85% of the subjects initially added to the groups	√	x	√
9. All subjects for whom outcome measure were available received the treatment or control condition as allocated or, where this was not the case, data for at least one key outcome was analysed by “intention to treat”	√	√	√
10. The results of between-groups statistical comparisons are reported for at least one key outcome	√	√	√
11. The study provides both point measures and measures of variability for at least one key outcome	√	√	√
Total	7/11	6/11	7/11

Criterion 3 (concealed allocation), criterion 5 (blinding of all subjects) and criterion 6 (blinding of the therapists) were not fulfilled in any of the three studies.

### Study sample description

Sample descriptions for each study is summarised in Table 2. Rawson et al. [28] included the largest sample, comprising 135 participants. All three articles specified the number of male and female individuals. Two studies were conducted in the USA [27, 28] and the third in China. The included studies were all published after the year 2013.

**Table 2:** Study sample description

Study	Rawson et al. [28]	Dolezal et al. [27]	Zhu et al. [29]
Sample size			
Exercise group	69	15	30
Control group	66	14	30
Gender			
Exercise group	Male (%): 70.4	Male (n): 13	Male (n): 30
	Female (%): 29.6	Female (n): 2	
Control group	Male (%): 70.4	Male (n): 12	Male (n): 29
	Female (%): 29.6	Female (n): 2	
Age (years)			
Exercise group	Mean ± SD: 31.7 ± 6.9	Mean ± SD: 30 ± 7	Mean ± SD: 37.47 ± 8.41
Control group	Mean ± SD: 31.7 ± 6.9	Mean ± SD: 32 ± 7	Mean ± SD: 41.69 ± 11.37
Acute/chronic symptoms at baseline	Not specified	Not specified	Not specified
Country	USA	USA	China

*n* number of participants, *SD* standard deviation, *USA* United Stated of America

### Description of intervention and control

A summary of the description of the intervention and control used in each article is presented in Table 3.

**Table 3:** Description of intervention and control procedures

Study	Rawson et al. [28]	Dolezal et al. [27]	Zhu et al. [29]
Exercise group			
Type of intervention	Warm-up, aerobic activity on a treadmill, resistance training with weightlifting, and cool-down with stretching	Endurance training: 1. Walked and/or jogged on a treadmill Resistance training for all major upper- and lower limb muscle groups: 2. Circuit-type weight training with selected machines 3. Dumbbell training	Tai Chi movements: Ye Ma Fen Zong (“part wild horse’s mane”), Dao Juan Gong (arm curls), Shou Hui Pi Pa (“play the flute”), Nan Que Wei (“grasping the peacock’s tail”), Yun Shou (“wave hands like clouds”), Jing Ji Du Li (“golden rooster stands on one leg”) and Dan Bian (“single whip”)
Frequency and duration	Fifty-five minutes in total: 5-min warm-up, 30 min aerobic activity, 15 min weight training and 5 min cool-down Exercises were scheduled 3 days per week for 8 weeks	One-hour sessions: 30 min of endurance training (walking and/or running) and approximately 30 min of resistance exercises, starting with one set of very light weights (8–15 reps) and progressing to two sets in the final 5 weeks; three times per week for 8 weeks	Total of 50 min: 10 min warm-up, 30 min Tai Chi and 10 min cool down Tai Chi exercise was scheduled five times per week over a period of 12 weeks
Control group			
Type of intervention	CBT: Health education sessions covering various health topics that included stress reduction, health screening, healthy relationships and sexually transmitted diseases	Education attention: Small-group health and wellness education. Material consisted of an integrated multimedia program addressing a variety of health-, wellness-, and lifestyle topics such as healthy eating, dental care, acupressure and cancer screening	Standard care included recreational activities, gesture language exercise (upper limb exercise with background music) and self-studying
Frequency and duration	Fifty-five-minute sessions, 3 days per week for 8 weeks	Wellness education sessions, presented three times per week, for about 1 h	Total of 50 min: 5 min recreational activities, 5 min gesture language exercise and about 40 min of self-study

*Reps* repetitions, *CBT* cognitive behavioural therapy

### Description of outcome measures

Two studies [27, 29] measured fitness as an outcome. Dolezal et al. [27] assessed fitness at the end of an 8-week program, via VO2max, body fat, body weight, fat weight, fat-free weight, 1-RM chest- and leg press, and 85% of 1-RM chest- and leg press. Zhu et al. [29] assessed fitness at the end of a 12-week program, by measuring body fat, BMI, blood pressure, heart rate, vital capacity, hand-grip, the Sit-And-Reach test and one-leg stand with eyes closed.

Quality of life was assessed by only one study, Zhu et al. [29], by administering the QOL-DA v2.0 at the final assessment at 12-weeks.

Anxiety and depression were assessed by only Rawson et al. [28], who performed assessments at baseline and at the end of an 8-week program. Anxiety was measured using the Beck Anxiety Inventory and depression using the Beck Depression Inventory.

### The effect of exercise compared to CBT, education and/or standard care

The effect of exercise on anxiety, depression, fitness and quality of life in adults with previous MA dependency, compared to CBT, education or standard care, is discussed under the subsequent subheadings:

#### Anxiety

Rawson et al. [28] found that the level of anxiety in the exercise group was significantly lower (p = 0.001) at the 8-week follow-up, compared to the CBT group. The exercise group’s anxiety scores changed from (mean ± SD) 16.5 ± 6.0 to 2.18 ± 4.94 at the 8-week follow-up, compared to the CBT group, whose scores changed from (mean ± SD) 11.9 ± 5.1 to 5.11 ± 7.79. Baseline scores were not statistically different.

#### Depression

Rawson et al. [28] recorded the level of depression in the exercise group to be significantly lower (p = 0.001) at the 8-week follow-up, compared to the CBT group. The exercise group’s anxiety scores changed from (mean ± SD) 13.7 ± 5.3 to 2.43 ± 4.22 at the 8-week follow-up, compared to the CBT group, whose scores changed from (mean ± SD) 12 ± 6.3 to 4.82 ± 5.71. Baseline scores were not statistically different.

#### Fitness

Zhu et al. [29] used body fat, BMI, blood pressure, heart rate, vital capacity, hand-grip, the Sit-And-Reach test, and one-leg stand with eyes closed as measures of fitness in MA dependent individuals. Balance improved significantly in the Tai Chi exercise group compared to the standard care group (p < 0.001); as did vital capacity, right and left hand-grip, and one-leg stand with eyes closed. Table 4 summarises the mean score-differences for each study group as well as the specific significance values.

**Table 4:** Mean differences ± SD in fitness measures between Tai Chi and standard care after 8 weeks in Zhu et al. [29]

Fitness measure	Tai Chi	Standard care group	p value
BMI (kg m^−2^)	0.80 ± 0.62	0.80 ± 0.58	0.288
Blood pressure (mmHg): systolic/diastolic	− 1.70 ± 15.96/− 6.97 ± 11.17	− 7.03 ± 15.23/− 11.90 ± 11.78	0.956/0.874
Heart rate (bpm)	5.20 ± 8.03	6.55 ± 8.62	0.530
Vital capacity (ml)	183.77 ± 831.60	197.45 ± 1092.44	0.026*
Hand-grip (right) (kgf)	0.39 ± 4.28	− 0.19 ± 3.99	0.006*
Hand-grip (left) (kgf)	0.41 ± 3.86	0.48 ± 4.41	0.001*
Sit-And-Reach test (cm)	− 0.43 ± 4.98	0.03 ± 5.04	0.548
One-leg stand with eyes closed (s)	10.34 ± 18.93	− 1.22 ± 8.99	0.002*

*BMI* body mass index; *bpm* beats per minute; *cm* centimetres*; kg* kilograms*; kgf* kilogram-force; *m* meter*; ml* millilitres; *mmHg* millimetres of mercury; *s* seconds; *SD* standard deviation

*Statistical significance

Dolezal et al. [27] used VO2max, body fat, weight, fat weight, fat-free weight, 1-RM chest press, 1-RM leg press and 85% of 1-RM chest- and leg press to measure fitness levels in MA dependent individuals. The exercise group demonstrated significant improvements when compared to equal attention (control) group after 8 weeks of intervention. Statistically significant differences were found for all fitness measures except fat-free weight (p = 0.344). Table 5 summarises the changes from baseline between the exercise and control groups.

**Table 5:** Mean differences ± SD of scores for exercise and equal-attention groups after 8 weeks in Dolezal et al. [27]

Fitness measure	Physical exercise (change)	Equal-attention (change)	p value
VO2max (l/min)	0.63 ± 0.06	− 0.02 ± 0.05	< 0.001*
Body fat (%)	− 2.8 ± 0.3	0.7 ± 0.4	< 0.001*
Weight (kg)	− 1.7 ± 0.6	1.7 ± 0.6	0.032*
Fat weight (kg)	− 2.8 ± 0.5	1.0 ± 0.4	< 0.001*
Fat-free weight (kg)	1.6 ± 0.5	0.6 ± 0.2	0.344
1-RM chest press (kg)	20.6 ± 1.5	1.3 ± 0.7	< 0.001*
1-RM leg press (kg)	24.4 ± 1.4	− 0.2 ± 0.6	< 0.001*
85% of 1-RM chest press (reps)	7.4 ± 0.3	− 0.1 ± 0.2	< 0.001*
85% of 1-RM leg press (reps)	9.7 ± 0.9	0.6 ± 0.3	< 0.001*

*1*-*RM* one-repetition maximum effort; *kg* kilograms; *l/min* litres per minute; *reps* repetitions; *VO2Max* maximum oxygen consumption/maximum aerobic capacity

*Statistical significance

#### Quality of life

Zhu et al. [29] assessed quality of life in MA dependent individuals using the QOL-DA, a scale including questions related to physiology, psychology, symptoms and society. Statistically significant changes were reported for all quality of life domains assessed by the questionnaire (p < 0.05), except psychology (p = 0.227). Results for quality of life are summarised in Table 6.

**Table 6:** Mean differences ± SD comparing Tai Chi versus standard care groups after 12 weeks for four domains of quality of life in Zhu et al. [29]

Quality of life domain	Tai Chi	Standard care	p value
Physiology	3.07 ± 5.59	− 1.07 ± 3.86	0.005*
Psychology	1.33 ± 7.6	− 1.14 ± 3.66	0.227
Symptoms	3.13 ± 8.98	− 1.03 ± 3.96	0.042*
Society	4.13 ± 6.57	− 1.03 ± 3.21	< 0.001*
Total scores	11.67 ± 21.57	− 4.79 ± 11.84	< 0.002*

*SD* standard deviation

*Statistical significance

## Discussion

The objective of this systematic review was to evaluate the effectiveness of exercise for reducing anxiety and depression, and improving fitness and quality of life in adults who have previously used MA. Our findings suggest that overall recovery in MA dependent users might be improved by including an effective exercise program in the rehabilitation process of these individuals. Though overall recovery was not specifically measured, an indirect improvement can be reasonably deduced from the reported outcomes, since significant improvements were found for anxiety, depression, fitness and quality of life following exercise, as compared to all control groups.

### Anxiety and depression

Individuals who have previously abused MA, with subsequent dysphoric mood symptoms, are prone to social isolation, suicidal thoughts and low socio-economic productivity [29], decreasing their quality of life [31] and increasing the public health burden [32]. Rawson et al. [28] demonstrated that exercise significantly reduces anxiety and depression symptoms associated with MA abstinence (p = 0.001), with an observed dose–response effect. Albeit from a single RCT, these results provide promising evidence for the key role of exercise in improving anxiety and depression, and indirectly quality of life, in previously MA-dependent adults.

Dysphoric mood symptoms are also associated with relapse and early treatment termination [33], to the extent that many individuals who have abused MA admit that they do not know anyone who has completed MA treatment [34]. Zhu et al. [29] found that the relaxation associated with Tai Chi reduces anxiety by helping individuals to become more self-aware and internally focused. Rawson et al. [28] agrees that exercise reduces mood-related symptoms of anxiety. Due to the benefits of exercise, along with the positive social influence participation in exercise groups may have on the individual, MA dependent adults may thus be more likely adhere to treatment and experience improved rehabilitation outcomes.

The exact mechanism through which exercise improves mood symptoms require further investigation [28], but several hypotheses are proposed. The manifestation of depression and anxiety in MA users results from the drug’s effect on the neuropathways; specifically, a decrease in dopamine-serotonin binding, making it impossible to experience pleasure naturally [9]. Furthermore, MA abuse increases the level of inflammatory markers whilst decreasing circulation [9]. Exercise, on the other hand, has been proposed to normalise dopamine-serotonin reactions [7], improve circulation and decrease the level of inflammatory biomarkers in the circulatory system [13]. In addition, aerobic exercise also increases the release of brain-derived neurotrophic factor (BDNF) [35]. BDNF, a neurotrophin, is responsible for neurogenesis and subsequently decreases long-term basal levels of cortisol [35, 36]. These changes decrease stress levels and subsequently reduce depression and anxiety [35]. Considering the burden that MA-associated mood disorders place on the individual, society and the healthcare system, and the low cost and simplicity of exercise as an intervention [37], implementation of exercise to improve mental and psychological well-being, and subsequent physical wellness, is warranted.

### Fitness

Postural balance is an essential component of motor and executive function. It is a particularly important outcome to address in MA users, given the BMI-loss, muscle wastage and neurological depression associated with the lethargic lifestyle typical to MA dependence and the direct effects of the drug on the brain [10]. Zhu et al. [29] demonstrated that Tai Chi, a moderate form of exercise, improved balance significantly more than standard care (p < 0.001), with a significantly improved performance of one leg standing with eyes closed (p = 0.002). Retraining functional balance using activities such as one leg standing in an individual suffering from postural instability as a result of drug use might be valuable to promote safety within the community, since postural balance impairments have been associated with unstable gait patterns and/or falls in several chronic and/or cognitive conditions, including drug abuse [38, 39]. Furthermore, improving balance might contribute to safe return to work, since intact balance is required for most functional activities [10]. Including balance-retraining exercises in the rehabilitation of previously MA-dependent individuals thus seems important to improve overall function. As trained specialists, physiotherapists are highly qualified in exercise administration as well as balance retraining, and should seize the opportunity to become more involved in the multi-disciplinary rehabilitation of those recovering from MA addiction.

Regarding vital capacity, Zhu et al. [29] demonstrated significant improvements (p = 0.026) for the intervention group, who performed moderate exercise in the form of Tai Chi, versus the control group. It is already a well-known fact that exercises improves vital capacity in various populations, yet the value of confirming this finding specifically in previous MA users should not be underestimated, considering the increased risk of chronic conditions such as hypertension and stroke [11] in this population. Considering that exercise prescription, functional mobility, cardiovascular- and cardiorespiratory training programs are well within their scope of practice, physiotherapists are in the ideal position to administer safe, supervised exercise programs as well as home programs within this population and thus have an important role to play in rehabilitative as well as preventative management during (and after) recovery.

Zhu et al. [29] suggested that exercise could lead to a normalization of BMI in previous MA users, although this finding was statistically non-significant. In clinical terms normalization of BMI would be important for this population, since chronic MA-use suppresses appetite, leading to eventual abnormal weight loss and below-normal BMI. This, in turn, may lead to secondary complications, such as malnourishment, starvation, decreased bone density, a weakened immune system, anaemia, hair loss, dry skin and infertility [11]. In addition to the obvious impact these complications in isolation may have on quality of life, their combined effects with other MA-associated co-morbidities should be considered—for example, balance impairments in individuals with low bone mineral density might lead to falls and fractures. Given the known beneficial effects of exercise on muscle mass, strength and function [40], it seems a feasible strategy for treating the complications associated with reduced BMI and there are thus grounds for further research.

### Quality of life

MA abuse is associated with withdrawal from social interaction and there is a need for strategies to promote community reintegration [41], possibly reducing quality of life. Exercise have been reported to improve self-esteem and cognitive function, and to alleviate social withdrawal [42], thereby improve quality of life. Zhu et al. [29] reported that exercise has a significant effect on almost all aspects of quality of life in previous MA users as measured by the QOL-DA. This finding supports the use of exercise among rehabilitating MA users to enhance quality of life and social interaction. Furthermore, exercise performed in group settings provides a platform for participants to interact with each other and improve social well-being [43]. The resultant mutual interaction and motivation amongst previously MA-dependent individuals may gradually increase during the intervention program, eventually cultivating self-efficacy, as was found in a study evaluating a 12-week Tai Chi program in adults with cardiovascular disease risk factors [44]. Future research is required to confirm and substantiate similar findings in previously MA-dependent adults and to inform future rehabilitation strategies.

### Limitations of included studies

Dolezal et al. [27] and Zhu et al. [29] included small sample sizes and were mainly based in residential settings, restricting extrapolation of findings to other MA populations. Future studies should include larger and more diverse populations to ensure wider generalisability. No blinding of assessors in Dolezal et al. [27] and Rawson et al. [28] was performed, which might have led to measuring bias. Although it is not always possible to blind both the therapist and participants in therapeutic trials, it is advised that at least the assessors should be blinded [45]. A further limitation was that the instrument used to assess quality of life in Zhu et al. [29], namely the QOL-DA v2.0, was previously only used for opioid-dependent individuals and not specifically for MA-dependent individuals. Outcome measures should be validated in specific populations to ensure that reliable data is collected [46]. None of the studies evaluated the long-term effect of exercise on anxiety, depression, quality of life and fitness, and future studies are therefore encouraged to include long-term follow up.

### Limitations of this systematic review

Only two appropriate RCTs and one quasi-experimental study were found eligible for inclusion in this review, due to the limited research available regarding the research question. Ideally, the results of a larger number of RCTs would be included and synthesized to generate a more valid conclusion. Substantial heterogeneity amongst the studies regarding outcome measures, assessment intervals and control groups prevented pooling of results of any of the included studies. This review furthermore excluded all non-English studies, increasing the risk for language bias. Lastly, it has to be acknowledged that the review process involved the exclusion of articles at title level which may inadvertently have excluded relevant studies, although the process was developed to reduce this possibility.

### Strengths of this systematic review

A comprehensive systematic search strategy was performed using seven databases, and results at each step of the consequent methodology were cross-check amongst at least two or more reviewers. All included articles were critically appraised using the PEDro scale and achieved high scores, thus increasing the reliability of the results of this review. The existence of good quality evidence presented in well-conducted systematic reviews provides clinicians with opportunities to better incorporate evidence-based practice, and alerts researchers to gaps in the literature.

### Recommendations for clinicians

Previous MA users are in need of a multidimensional rehabilitation approach that includes exercise, to promote overall well-being and reintegration into society, and reduce the risk of relapse and subsequent adverse events. In the light of the promising findings of this systematic review regarding the various benefits of exercise in this specific population, healthcare professionals, especially physiotherapists, need to be educated and encouraged to implement exercise programs for previously MA-dependent individuals. This will not only positively change the individual lives of previous MA users, but may also have a positive influence on their families and the wider community—as these individuals will have a greater chance of successful rehabilitation, enabling them to participate in and contribute to society. The role of physiotherapists and other health professionals in the successful management and social reintegration of previously MA-dependent adults needs to be further established by future research and emphasized amongst practitioners to ensure a more holistic and comprehensive approach to this societal dilemma.

## Conclusion

In conclusion, the aim of this systematic review was to establish the effect of exercise on anxiety and depression symptoms, fitness and quality of life experienced by previously MA-dependent adults. Level II evidence suggests that exercise is effective in reducing anxiety and depression and improving fitness in previous MA users, and Level III-2 evidence (ranked according to the NHMRC hierarchy of evidence) suggests that exercise is beneficial for improving quality of life in this population. The findings of this review thus indicate that the overall recovery in MA dependents might be significantly enhanced by including an effective exercise program to the rehabilitation process. Implementing such programs might not only positively change the lives of affected MA dependents, but may also influence the wider community and decrease strain on public healthcare. Further research is required to strengthen these conclusions and to inform policy and health systems effectively.

## Additional files

**Additional file 1.** Search strategy. Detailed search strategies, specifically developed for each database according its functions.

**Additional file 2.** The National Health and Medical Research Council (NHMRC) Evidence Hierarchy. The NHMRC Evidence Hierarchy summarised in tabular form.

**Additional file 3.** The PEDro scale. The PEDro scale, including notes on administration (used for methodological appraisal of included studies).

**Additional file 4.** Adapted Joanna Briggs Institute Data Extraction Form. Data extraction form, adapted from the standardized Joanna Briggs Institute data extraction form for the purposes of this review.

## Abbreviations

1-RM: one-repetition maximum effort
BAI: Beck Anxiety Inventory
BDI: Beck Depression Inventory
BDN: brain-derived neurotrophic factor
BMI: body mass index
bpm: beats per minute
CBT: cognitive behavioural therapy
cm: centimeters
IFNα: interferon alpha
IFNβ: interferon beta
JBI: Joanna Briggs Institute
kg: kilograms
Kgf: kilogram-force
m: meters
MA: methamphetamine
ml: milliliters
mmHg: millimeters of mercury
QOL-DA: Quality of Life for Drug Addiction Questionnaire
RCT: randomised controlled trial
s: seconds
SD: standard deviation
USA: United States of America
VO2Max: maximum oxygen consumption/maximum aerobic capacity
