# Supplementary material for: Effect of exercise versus cognitive behavioural therapy or no intervention on anxiety, depression, fitness and quality of life in adults with previous methamphetamine dependency: a systematic review
Source: Addict Sci Clin Pract. 2018 Jan 16;13:4. doi: 10.1186/s13722-018-0106-4 (PMC5771022; doi:10.1186/s13722-018-0106-4)
Supplement: Supplementary file 1 — Additional file 1. Search strategy. Detailed search strategies, specifically developed for each database according its functions. [file 13722_2018_106_MOESM1_ESM.pdf]

Additional file 1: Search Strategy

Pubmed

Limits applied to the database

Type of search: Simple search

Publication dates: Inception to May 2017

Publication types: Randomised controlled trials

Population: Humans

Language: English

Search Terms

1.	Methamphetamine
2.	exercise OR physical activity
3.	#1 AND #2
4.	depression OR depression outcomes
5.	#3 AND #4
6.	fitness measures
7.	#3 AND #6

Cochrane Library

Limits applied to the database

Type of search: Advanced search

Publication type: Randomised controlled trials

Publication dates: Inception to May 2017

MeSH terms

"Methamphetamine" [MeSH] AND "exercise" [MeSH]

Search Terms

1.	Methamphetamine [MeSH Major topic]
2.	exercise [MeSH]
3.	#1 AND #2
4.	depression [MeSH]
5.	anxiety [MeSH]
6.	#3 AND #4
7.	#3 AND #5
8.	fitness measures
9.	#3 AND #8

Science Direct

Limits applied to the database

Type of search: Simple and advanced search

Publication dates: Inception to May 2017

Search Terms

1.	Methamphetamine
2.	exercise OR physical activity
3.	#1 AND #2
4.	depression
5.	anxiety
6.	psychological disorders
7.	#3 AND #4
8.	#3 AND #5
9.	#3 AND #6
10.	fitness measures
11.	#3 AND #10

Ebsco Host: CINAHL, pre-CINAHL

Limits applied to the database

Type of search: Advanced search

Publication dates: Inception to May 2017

Publication type: Randomised controlled trials

Language: English

Search Terms

1. Methamphetamine
2. exercise OR physical activity
3. #1 AND #2
4. depression
5. anxiety
6. psychological disorders
7. #3 AND #4
8. #3 AND #5
9. #3 AND #6
10. fitness measures
11. #3 AND #10

PEDro

Limits applied to the database

Type of search: Advanced search

Publication dates: Inception to May 2017

Publication type: Randomised controlled trials

Randomised review

Search Terms

1. Methamphetamine
2. exercise OR physical activity
3. #1 AND #2
4. depression

5.	anxiety
6.	psychological disorders
7.	#3 AND #4
8.	#3 AND #5
9.	#3 AND #6
10.	fitness measures
11.	#3 AND #10

Scopus

Limits applied to the database

Type of search: Advanced search

Publication dates: Inception to May 2017

Publication type: Randomised controlled trials

Language: English

Search Terms

1.	Methamphetamine
2.	exercise OR physical activity
3.	#1 AND #2
4.	depression
5.	anxiety
6.	psychological disorders
7.	#3 AND #4
8.	#3 AND #5
9.	#3 AND #6
10.	fitness measures
11.	#3 AND #10

MEDLINE - ProQuest

Limits applied to the database

Type of search: Advanced search

Publication dates: Inception to May 2017

Publication type: Randomised controlled trials

Language: English

Search Terms

1.	Methamphetamine [MeSH Major topic]
2.	exercise [MeSH]
3.	#1 AND #2
4.	depression
5.	anxiety
6.	psychological disorders
7.	#3 AND #4
8.	#3 AND #5
9.	#3 AND #6
10.	fitness measures
11.	#3 AND #10
