# Supplementary material for: Effect of exercise versus cognitive behavioural therapy or no intervention on anxiety, depression, fitness and quality of life in adults with previous methamphetamine dependency: a systematic review
Source: Addict Sci Clin Pract. 2018 Jan 16;13:4. doi: 10.1186/s13722-018-0106-4 (PMC5771022; doi:10.1186/s13722-018-0106-4)
Supplement: Supplementary file 2 — Additional file 2. The National Health and Medical Research Council (NHMRC) Evidence Hierarchy. The NHMRC Evidence Hierarchy summarised in tabular form. [file 13722_2018_106_MOESM2_ESM.pdf]

Additional file 2: The National Health and Medical Research Council (NHMRC) Evidence Hierarchy

Level	Intervention
I	A systematic review of level I studies
II	A randomized control trial
III - 1	A pseudo-randomized controlled trial (i.e. alternate allocation or some other method)
III - 2	A comparative study with concurrent controls:<br>1. Non randomized, experimental trial<br>2. Cohort study<br>3. Case – control study<br>4. Interrupted time series with a control group
III-3	A comparative study without concurrent controls:<br>1. Historical control study<br>2. Two or more single arm study<br>3. Interrupted time series without a parallel control group
IV	Case series with either post-test or pre–test/post–test outcomes

Merlin T, Weston A, Tooher R. Extending an evidence hierarchy to include topics other than treatment: revising the Australian "levels of evidence". BMC Med. Res. Methodol. 2009;9:34.
