# Supplementary material for: Effect of exercise versus cognitive behavioural therapy or no intervention on anxiety, depression, fitness and quality of life in adults with previous methamphetamine dependency: a systematic review
Source: Addict Sci Clin Pract. 2018 Jan 16;13:4. doi: 10.1186/s13722-018-0106-4 (PMC5771022; doi:10.1186/s13722-018-0106-4)
Supplement: Supplementary file 4 — Additional file 4. Adapted Joanna Briggs Institute Data Extraction Form. Data extraction form, adapted from the standardized Joanna Briggs Institute data extraction form for the purposes of this review. [file 13722_2018_106_MOESM4_ESM.pdf]

Additional file 4: Adapted Joanna Briggs Institute Data Extraction Form

CITATION			
**Database:**			
**Authors:**			
**Title:**			
**Publication date:**			
**PEDro Score:**			
**Journal:**	Journal:		
	Volume:	Issue:	Page numbers:
**Thesis / Dissertation:**	Institution:		
**Country where research was conducted:**			

TYPE OF STUDY
☐ Randomised Controlled Trial
☐ Quasi-randomised Controlled Trial
☐ Controlled Clinical Trial
☐ Other:

PARTICIPANTS			
**Number of participants:**	Total:	Completed study:	Withdrawn:
**Gender**:	Total ♂:	Total ♀:	
**Randomized**:	☐ Yes	☐ No	
**Mean age:**			

INTERVENTION			
**Intervention:** Educational-behavioural joint protection education	Description:		
**Comparison:** Conventional joint protection education	Description:		
**Interventions adequately described:**	☐ Yes	☐ No	☐ Not clear

OUTCOME MEASURES			
**Outcome:**	**Measurement tool:**	**Validity:**	**Reliability:**
☐ Pain			
☐ Function			
☐ Quality of life			
☐ Other:			

RESULTS: DICHOTOMOUS DATA				
**Outcome:**	**Intervention Group:** Educational-behavioural joint protection education		**Comparison Group:** Conventional joint protection education	
	**Short term**	**Long term**	**Short term**	**Long term**
1.				
2.				
3.				
4.				
5.				

RESULTS: CONTINUOUS DATA				
**Outcome:**	**Intervention Group:** Educational-behavioural joint protection education **Mean and SD (number)**		**Comparison Group:** Conventional joint protection education **Mean and SD (number)**	
	**Short term**	**Long term**	**Short term**	**Long term**
1.				
2.				
3.				
4.				
5.				

CLINICAL STATUS AND IMPLICATION (AUTHOR'S CONCLUSIONS)			
**Clinical status post intervention:**	☐ Improved	☐ Deteriorated	☐ Consistent
**Clinical implication:**			
**Author's conclusions:**			
